# Characterization of the Skin Microbiota of the Cane Toad *Rhinella cf. marina* in Puerto Rico and Costa Rica

**DOI:** 10.3389/fmicb.2017.02624

**Published:** 2018-01-05

**Authors:** Juan G. Abarca, Ibrahim Zuniga, Gilmary Ortiz-Morales, Armando Lugo, Mariel Viquez-Cervilla, Natalia Rodriguez-Hernandez, Frances Vázquez-Sánchez, Catalina Murillo-Cruz, Ernesto A. Torres-Rivera, Adrián A. Pinto-Tomás, Filipa Godoy-Vitorino

**Affiliations:** ^1^Centro de Investigación en Estructuras Microscópicas, Universidad de Costa Rica, San Pedro de Montes de Oca, Costa Rica; ^2^Departamento de Bioquímica, Escuela de Medicina, Universidad de Costa Rica, San Pedro de Montes de Oca, Costa Rica; ^3^Centro de Investigación en Biología Celular y Molecular, Universidad de Costa Rica, San Pedro de Montes de Oca, Costa Rica; ^4^Department of Natural Sciences, Microbial Ecology and Genomics Laboratory, Inter American University of Puerto Rico, Metropolitan Campus, San Juan, Puerto Rico; ^5^Department of Natural Sciences, Center for Environmental Education, Conservation and Interpretation, Inter American University of Puerto Rico, Metropolitan Campus, San Juan, Puerto Rico

**Keywords:** 16S rRNA gene sequencing, skin, toad, bacterial communities, bioinformatics

## Abstract

*Rhinella marina* is a toad native to South America that has been introduced in the Antilles, likely carrying high loads of microorganisms, potentially impacting local community diversity. The amphibian skin is involved in pathogen defense and its microbiota has been relatively well studied, however, research focusing on the cane toad microbiota is lacking. We hypothesize that the skin microbial communities will differ between toads inhabiting different geographical regions in Central America and the Caribbean. To test our hypothesis, we compared the microbiota of three populations of *R. cf. marina* toads, two from Costa Rican (native) and one Puerto Rican (exotic) locations. In Costa Rica, we collected 11 toads, 7 in Sarapiquí and 4 from Turrialba while in Puerto Rico, 10 animals were collected in Santa Ana. Separate swab samples were collected from the dorsal and ventral sites resulting in 42 samples. We found significant differences in the structure of the microbial communities between Puerto Rico and Costa Rica. We detected as much as 35 different phyla; however, communities were dominated by Proteobacteria, Bacteroidetes, Firmicutes, and Actinobacteria. Alpha diversity and richness were significantly higher in toads from Puerto Rico and betadiversity revealed significant differences between the microbiota samples from the two countries. At the genus level, we found in Santa Ana, Puerto Rico, a high dominance of *Kokuria*, *Niabella*, and Rhodobacteraceae, while in Costa Rica we found *Halomonas* and *Pseudomonas* in Sarapiquí, and *Acinetobacter* and *Citrobacter* in Turrialba. This is the first report of *Niabella* associated with the amphibian skin. The core microbiome represented 128 Operational Taxonomic Units (OTUs) mainly from five genera shared among all samples, which may represent the symbiotic *Rhinella*’s skin. These results provide insights into the habitat-induced microbial changes facing this amphibian species. The differences in the microbial diversity in Puerto Rican toads compared to those in Costa Rica provide additional evidence of the geographically induced patterns in the amphibian skin microbiome, and highlight the importance of discussing the microbial tradeoffs in the colonization of new ecosystems.

## Introduction

In the last 30 years amphibians have undergone massive population declines (Whittaker et al., 2013). This phenomenon is attributed to climate change, habitat loss, pollution, and the presence of emerging infectious diseases, among other causes (Whitfield et al., 2016). It is suggested that the appearance of these emerging diseases is due to the introduction of exotic pathogens, such as *Batrachochytrium dendrobatidis* (*Bd*) (Longcore et al., 1999), Ranavirus (Price et al., 2014) or, more recently, *B. salamandrivorans* (*Bsal*), a fungus that affects salamanders (Martel et al., 2013). Pathogen spread has also been attributed to human trafficking of amphibian species (Bacigalupe et al., 2017). Due to the increase of infectious diseases, introduced species represent a constant threat to local fauna (Schloegel et al., 2009). Problems with introduced amphibians and reptiles have occurred worldwide, as in the case of the bullfrog (*Lithobates catesbeianus*) in the western areas of the United States, the Caribbean, and in South America (Young et al., 2004), the brown tree snake (*Boiga irregularis*) on Guam Island (Savidge et al., 2007), and also the giant toad or cane toad (*Rhinella cf. marina*) in Australia (Shine, 2010). In Puerto Rico, a decline of several native amphibian species has been documented, and among other possible factors is the introduction of the pathogen *Bd*, drought, and habitat loss (Burrowes et al., 2004). In addition, Puerto Rico has a great number of introduced species maintaining a constant threat to the native fauna, including six species of frogs (Joglar et al., 2007). The cane toad is one of such species, introduced in Puerto Rico in the early 20th century aiming at controlling a beetle infestation in sugarcane plantations, successfully halting the damage (Tyler, 1989; Thomas, 1999).

The cane toad has, in fact, a broad geographic distribution. It is native to the United States (South Texas), Central America (including Costa Rica), and South America, including Trinidad and Tobago. In these places the cane toad is not a threat and its populations appear to be stable (Solís et al., 2009). Its history of invasiveness dates back to the 1800s when it was introduced in Barbados and Jamaica, in 1920 in Florida and Puerto Rico, in 1930–1935 in Philippines and Australia, respectively, and from there to Japan in 1978 including other islands (Solís et al., 2009). Many of these introductions have been made with the aim of controlling agricultural pests, but have had little proven success. The cane toad has become a constant threat and the Invasive Species Specialist group of the Union for Conservation of Nature (IUCN) has declared it one of the 100 most damaging invasive species in the world (Lowe et al., 2000). Recent taxonomic changes subdivided this species into *R. horribilis* for Central America and *R. marina* for South America (Acevedo et al., 2016); however, the taxonomic status of the introduced populations is not clear and more genetic analyses are needed to verify these changes (Acevedo, personal communication).

When introducing an exotic species, either accidentally or intentionally, the potential pathogens that can be loaded are generally not analyzed, because molecular microbiological essays are never performed. It has been documented that the cane toad can carry *Salmonella* species that can affect other native species (Burrowes et al., 2004), and pathogen transmission between the cane toad and other species has even been documented in Panamá (Kelehear et al., 2015). These pathogens can be a severe problem to local fauna since invasive species are difficult to control and eliminate. Furthermore, some frog species are much less susceptible to death from particular pathogens and may act as carriers; for example, the cane toad is less susceptible to *Bd* but can still carry it as asymptomatic infections (Lips et al., 2006).

It is now possible to study the diversity of microbial communities in any habitat or species through next-generation sequencing, an approach that has allowed researchers to characterize the patterns of changes in the microbiota, revealing possible pathogens and symbionts associated with a given host (Rebollar et al., 2016a). One such example is the resistance of some frogs to pathogens, likely due to the presence of beneficial bacteria in their skin (Harris et al., 2009). Culture-independent techniques have shown differences in bacterial diversity depending on the degree of *Bd* infection among the same amphibian species (Rebollar et al., 2016a,b).

Variations in the skin microbiota of species across different geographies have been attributed to several factors, including: (1) the selective force excerpted by the chytrid fungus Bd (Walke et al., 2015; Rebollar et al., 2016b), (2) additive and non-additive mechanisms underlying the dilution effect (Becker et al., 2014), (3) environmental factors and host genetics and ecology (Kueneman et al., 2014; Bletz et al., 2017a), or (4) environmental connectivity (Walke et al., 2014).

Even though there have been several reports on the microbiota of amphibians, there are no studies on the Cane toad skin microbiota (Jiménez and Sommer, 2017), despite its wide distribution and propensity for acting as a vector of infectious diseases, and the capability of biotransformation of their chemical defenses in their parotid glands (Kamalakkannan et al., 2017). Similarly, amphibian bacterial communities have been compared between families in temperate and tropical regions (Belden et al., 2015) but to the best of our knowledge there are no studies comparing the same species in two geographically distant regions.

To bridge this knowledge gap, this work represents the first report comparing the microbial communities of *R. cf. marina* toads in its native (Costa Rica) and exotic (Puerto Rico) ranges, a preliminary study on animals from two countries that share similar tropical ecosystems.

We hypothesize that there will be differences in the skin microbial communities between the dorsal and ventral sides of toads, and between the three sampling locations in its native (Costa Rica) and exotic (Puerto Rico) ranges. Here, we identify the differences between microbial communities of toads in Puerto Rico and Costa Rica, define the unique taxa for each locality, and define which bacterial groups compose the core microbiome of this species.

## Materials and Methods

### Cane Toad Sampling

Field sampling was conducted between July and October 2016 in La Selva (LS) Biological Station Sarapiquí, Costa Rica (10, 25.816 N, 84, 0.550 W; elev. ∼60 m); Turrialba City (TC), Costa Rica (9, 53.897 N, 84, 40.330 W; elev. 600 m); and Centro Ambiental Santa Ana (SA) Bayamón, Puerto Rico (18, 24.480 N, 66, 8.651 W; elev. 20–60 m). Here we applied the Holdridge classification system (Holdridge, 1967) that considers tropical altitudinal height to be in a range of 0–700 m. A total of 21 Cane toads were collected using disposable nitrile gloves. Each toad was washed for 7 s using 50 ml of sterile distilled water to reduce transient surface bacteria (Madison et al., 2017). Sterile swabs were rubbed 10 times in the ventral and the dorsal area of toad, yielding two samples per individual. This study was exempt from IACUC protocol review since animals were collected without interfering with its environment. After the brief sterile skin swabbing *in situ*, toads were released immediately in their natural environment.

The swabs were placed in labeled Power Bead tubes (MoBio PowerSoil DNA Extraction Kit) into a cryobox in an ice-filled container and transported to the laboratory for -80°C storage. A total of 42 swab samples were obtained from the ventral and dorsal skin surfaces of toads, 20 from Puerto Rico and 22 from Costa Rica. For each individual toad, we measured the following parameters: skin surface pH in the dorsal area with a universal paper strip (Hydrion Paper); length and width employing a caliper, toads were placed inside the collection bag and weighed using a scale (Hanson). All sampled individuals were adults, although those from Sarapiquí Costa Rica were young adults. Environmental variables including temperature, humidity, and precipitation were obtained from nearby meteorological stations in both countries.

### DNA Extraction

Genomic DNA was extracted from the swab material using the PowerSoil DNA Isolation Kit (MO BIO, Carlsbad, CA, United States) following the manufacturer’s instructions with the following modifications: (1) samples were incubated at 65°C after the addition of reagent C1; (2) the powerbead tubes were homogenized horizontally for 2 min at 2000 rpm, using a PowerLyzer^TM^ 24 Bench Top Bead-Based Homogenizer (MO BIO, Carlsbad, CA, United States); and (3) the elution buffer was allowed to sit on the filter for 5 min before the final centrifugation step.

To increase DNA yield, we used the pellet formed from the MO BIO powerbead for a second DNA extraction and pooled the two extractions per sample.

### 16S rRNA Gene PCR and Sequencing

The V4 hypervariable region of the 16S ribosomal RNA (∼291 bp length) was amplified by PCR using the universal bacterial and archaeal primers: 515F (5′-GTGCCAGCMGCCGCGGTAA-3′) and 806R (5′-GGACTACHVGGGTWTCTAAT-3′) as described in the Earth Microbiome Project (EMP^1^) (Caporaso et al., 2012) using the following amplification conditions: 1 cycle of 94°C for 3 min, and 35 cycles of 94°C for 45 s and 50°C for 60 s and 72°C for 90 s, and a final extension of 72°C for 10 min.

16S amplicons were sent to the Sequencing and Genotyping Facility of the University of Puerto Rico for sequencing with the Illumina MiSeq System MSQ-M00883. The resulting post QC good-quality sequences for each sample were deposited in the NCBI BioProject ID PRJNA391810.

### Sequence Processing and Data Analysis

A first quality control analyses using FastQC (Andrews, 2010) revealed that only forward reads were useful for downstream analyses. Sequences were de-multiplexed and processed using QIIME (Caporaso et al., 2010) with a Phred score of 20 and chimera filtering with the usearch61 hierarchical clustering method (Edgar et al., 2011). Sequences were clustered into Operational Taxonomic Units (OTUs) using *uclust* (Edgar, 2010) with a 97% identity threshold. Taxonomic assignment was performed using the RDP classifier with a minimum confidence threshold of 80%. Contaminant chloroplast and low abundance OTUs were removed from downstream analyses using the script filter_taxa_from_otu_table in QIIME (Kuczynski et al., 2012). Analyses were done in two ways: (1) considering both samples (dorsal and ventral) (*n* = 42) and (2) per individual, by collapsing ventral and dorsal samples in the BIOM table and mapping file into one data point using the -collapse_mode mean available in QIIME (*n* = 21). The resulting OTUs underwent rarefication to mitigate bias due to different sequence depth per sample. Values in the mapping file were also collapsed by grouping dorsal and ventral samples into one sample. The data analyses were done considering only those OTUs that were present in at least 50% of the samples; therefore, it eliminated much of the rare OTUs.

We used a QIIME diversity analyses workflow script core_diversity_analyses.py, for both alpha and beta diversity analyses for the main metadata categories of the mapping file country and location. The data analyses were performed using a rarefaction level of 3670 sequences per sample when considering all 42 samples (dorsal and ventral swabs), and of 32,900 sequences when collapsing dorsal and ventral samples in individuals, to avoid the bias caused by differences in sequence depth. This core diversity workflow does an extensive diversity analyses including alpha rarefaction diversity analyses such as the Chao 1 abundance-based richness estimator and the phylogenetic diversity (PD) metric of Faith, both computed in QIIME. Chao 1 values represent the estimated true species richness of a sample and are calculated with the script for alpha rarefaction in QIIME that in turn implements the Chao 1 abundance-based estimator (Chao, 1987). It also calculates the PD metric of Faith, which does not take abundance into account but rather branch lengths of the phylogenies connecting all species to each community (Faith, 1992). The alpha rarefaction on the OTU table alpha_diversity.py, results in many files, that are then concatenated into a single file for generating rarefaction curves (collated file) to which statistical tests were applied. The rarefaction plots were recreated using the R package *Hmisc* (Harrell, 2006) using the output results of the rarefaction curves in QIIME.

Beta diversity analysis was performed as a non-metric multidimensional scaling plot (NMDS) using the Bray–Curtis distance metric and calculating *stress values* using the R packages Phyloseq (McMurdie and Holmes, 2013), vegan (Oksanen et al., 2008), and ggplot2 (Wickham, 2009) through the *ordinate* function. The Bray–Curtis matrix was calculated on the OTU table using script beta_diversity.py with the metrics option bray_curtis.

Taxonomic summaries at the Phyla and Genus levels were built by using QIIME’s Taxa_Summary plot tables L2 and L6, respectively, using the *melt* function in the RESHAPE2 R package (Wickham, 2007). The significantly different phyla as determined by ANOVA, as well as the selected genus-level OTUs significantly associated with each location, were visualized as boxplots combining R packages ggplot2 (Wickham, 2009), RColorBrewer (Neuwirth, 2014), and scales (Wickham, 2017).

A heatmap of the significantly different taxa (FDR-adjusted *p*-values) for the two metadata categories (location and country) was built using heatmap.3 function in R (Zhao et al., 2015). Data normalization was done through DESeq2 negative binomial Wald normalization for visualization purposes due to differences in the numbers of individuals per sample. This normalization step was implemented in QIIME using the script normalize_table.py.

Additionally, the core microbiome was calculated for all samples using the compute_core_microbiome script in QIIME (Kuczynski et al., 2012) and the resulting OTU list was used to create a new OTU table used for plotting a Taxa Summary in QIIME (Kuczynski et al., 2012).

### Statistical Analyses

Metadata categories were compared between each site using one-way ANOVA in R (v. 3.2.5) (R Development Core Team, 2008).

Significant differences of alpha diversity were calculated using a non-parametric two-sample *t*-test using 999 Monte-Carlo permutations using the QIIME (Caporaso et al., 2010) script compare_alpha_diversity.py using the collated alpha diversity file resulting from the alpha rarefaction analyses. The comparison was in fact done not between samples, but between groups of samples, created via the input category passed via “-c” on the mapping file. Significance tests were computed for each group comparison with the Chao1 abundance-based estimator, the alpha PD metric of Faith, and the Shannon index, for the 42-sample dataset. Same significance tests on alpha PD and Chao 1 were used on the 21-sample dataset.

Statistical tests on the beta diversity were done via nonparametric PERMANOVA significance in QIIME (Caporaso et al., 2010) through compare_categories.py script. This PERMANOVA test is determined through permutations and provides strength and statistical significance on sample groupings using a Bray–Curtis distance matrix as the primary input.

We performed Analyses of Variance tests using the *aov*( ) function in R (R Development Core Team, 2008) on the abundance values at each taxonomic Phyla, using the -biom-derived data matrices from QIIME (L2 table), comparing the relative abundance of each Phyla in the three sampling locations. Boxplots of the significant changes at the phyla level were plotted with ggplot2 (Wickham, 2009) and RColorBrewer (Neuwirth, 2014), using a normalized table of values, by running the R interface package of DESeq2 for table normalization, DESeq outputs negative values for lower abundant OTUs as a result of its log transformation.

Significantly different OTUs across countries and locations were detected through a log-likelihood ratio test, that detects what OTUs changed significantly in relative abundance between the two countries and the three habitats (locations) using the *G*-test with QIIME’s group_significance script (Kuczynski et al., 2012), with the alternate hypothesis that the frequency of the OTUs would not be the same across all sample groups. Only FDR-adjusted *p*-values (*p* < 0.05) were taken in consideration.

## Results

A total of 5,296,165 good quality sequences were employed in the analyses. Among these, 1,967,761 sequences were obtained from Puerto Rican samples (Santa Ana) and they were binned into 3779 OTUs (**Table 1**). The Costa Rican samples included 1,296,254 sequences from Sarapiquí that were binned into 2253 OTUs and those from Turrialba in which 2,099,150 sequences were binned in 3516 OTUs (**Table 1** and **Supplementary Tables S2**, **S3**). **Supplementary Table S1** summarizes the number of sequences and OTUs for all 42 samples and **Supplementary Table S2** summarizes the number of sequences and OTUs for the 21 collapsed samples.

**Table 1:** Number of sequences and OTUs across samples.

Country (site/habitat)	Number of animals	Number of samples	Number of sequences	Average number of OTUs ± Stdev
Puerto Rico (Santa Ana)	10	20	1,967,761	3779 ± 840
Costa Rica (Sarapiquí)	7	14	1,229,254	2253 ± 1013
Costa Rica (Turrialba)	4	8	2,099,150	3516 ± 798


We compared differences in weight and pH among the two populations from which we had these values – Sarapiquí, Costa Rica, and Santa Ana, Puerto Rico, and found that animals in Sarapiquí weighed significantly less than those from Santa Ana (ANOVA, *df* = 1, *F*-value = 117.1, *p*-value = 1.76*e*-08), and their pH was also significantly higher (*df* = 1, *F*-value = 13.97, *p*-value = 0.00198). There were no significant differences in length between these animals although some of the individuals in Sarapiquí were smaller (**Supplementary Table S4**). Environmental measurements in the collection sites were very similar across the three locations, confirming that these sites have the same tropical environmental conditions in both countries.

**FIGURE 1:**
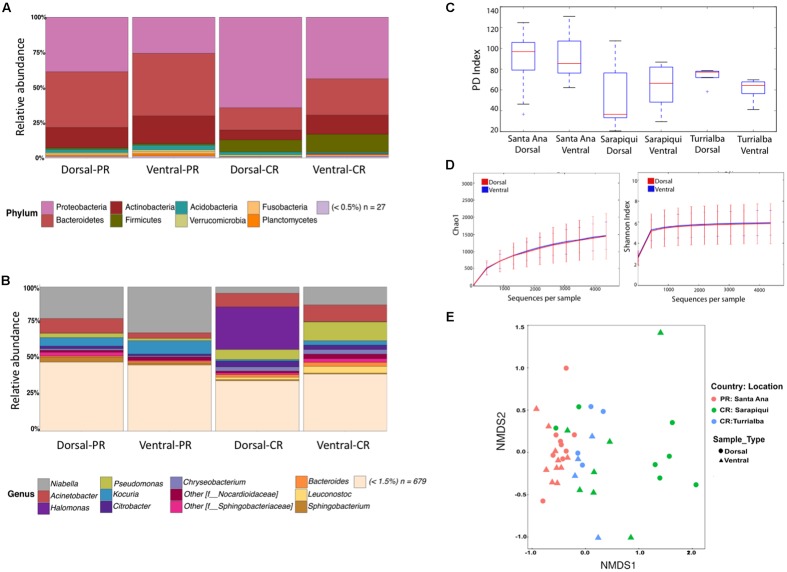
Microbiota diversity in dorsal and ventral swab samples among toads in Puerto Rico and Costa Rica. **(A)** Taxonomic bar plots showing bacterial phyla among ventral and dorsal samples. **(B)** Taxonomic bar plots at the genus level. **(C)** Faith’s phylogenetic diversity (PD) boxplots overall dorsal and ventral swabs per location. **(D)** Rarefaction plots of Chao1 (*t*-test, *t*-stat = –0.072, *p*-value = 0.94) and Shannon (*t*-test, *t*-stat = 0.164, *p*-value = 0.868) between dorsal and ventral skin sites. **(E)** Non-metric multidimensional scaling (NMDS) plots of samples according to location and sample type (stress = 0.15 and PERMANOVA Pseudo-*F*: 0.965, *p*-value = 0.461).

We found no significant differences between the microbial community structure in dorsal and ventral samples in any of the three locations (**Figure 1**). We found a total of 35 assigned phyla, with 6 of these dominating across all the samples: Proteobacteria, Bacteroidetes, Actinobacteria, Firmicutes, Acidobacteria, and Verrucomicrobia; with the other 29 phyla having a relative abundance lower than 1% (**Figure 1A**). Overall, at the genus level we found a dominance of *Niabella* and *Pseudomonas* across all samples (**Figure 1B**). The PD was nearly identical between ventral and dorsal swab samples at each of the three locations: Santa Ana dorsal vs. ventral (*t*-test, *t*-stat = 0.175, *p*-value = 1); Sarapiqui dorsal vs. ventral (*t*-test, *t*-stat = -0.477, *p*-value = 1), and Turrialba dorsal vs. ventral (*t*-test, *t*-stat = 1.591, *p*-value = 1) (**Figure 1C** and **Supplementary Table S4**). Rarefaction plots of Chao1 (*t*-test, *t*-stat = -0.072, *p*-value = 0.94) and Shannon (*t*-test, *t*-stat = 0.164, *p*-value = 0.868) confirm that there were no significant differences between dorsal and ventral skin sites (**Figure 1D**). Beta diversity comparisons between all 42 samples separated mostly samples from Turrialba (Costa Rica) from the rest, but did not separate ventral and dorsal samples (PERMANOVA, Pseudo-*F*: 0.9657, *p*-value = 0.461) (**Figure 1E** and **Supplementary Table S4**). As the analyses of the 42 samples did not show significant differences, we collapsed the dorsal and ventral samples considering now 21 samples, one per individual.

**FIGURE 2:**
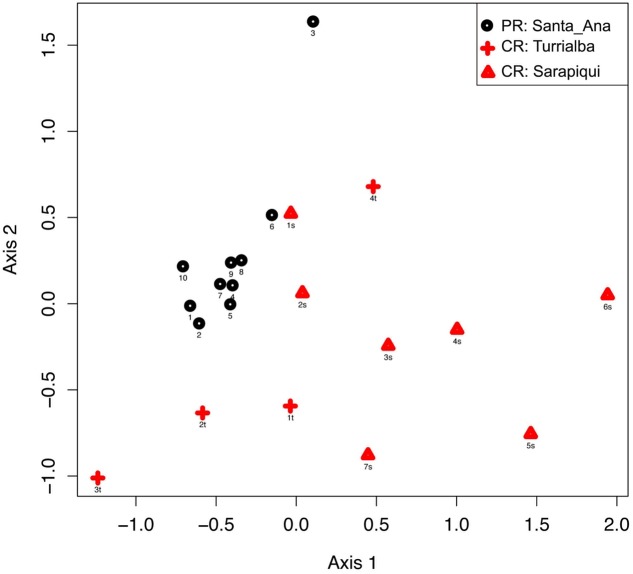
Beta diversity comparisons by NMDS, stress = 0.156. Ordinations of Bray–Curtis dissimilarity between the bacterial communities inhabiting the three different locations in the two countries show a clear separation by country (PERMANOVA, Pseudo-*F*: 5.05, *p*-value = 0.001) and by location (PERMANOVA, Pseudo-*F*: 4.65, *p*-value = 0.001).

Hence, considering the 21 individuals, microbial communities in the samples from Puerto Rico were clearly grouped together as shown by NMDS based on the relative dissimilarities of the samples (Bray–Curtis) with a stress value of 0.156. Costa Rican samples show a close aggregation with Puerto Rican samples, especially those from Turrialba (**Figure 2**). We found significant differences among microbial communities of the two countries (PERMANOVA, Pseudo-*F*: 5.05, *p*-value = 0.001); also, validated by an ANOSIM test (test statistic = 0.421 and *p*-value = 0.01). We also found the microbial communities in the three locations to be significantly different (PERMANOVA, Pseudo-*F*: 4.65, *p*-value = 0.001; **Supplementary Table S4**).

**FIGURE 3:**
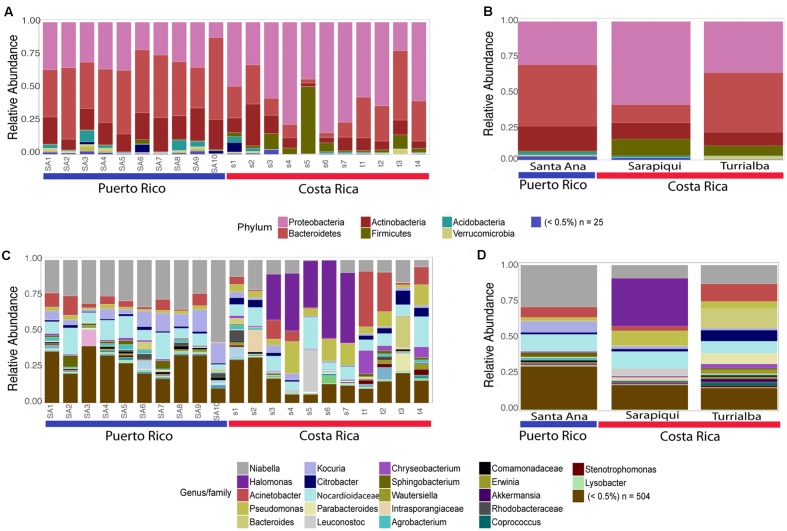
Taxonomic profiles at the phyla-level **(A,B)** and genus-level **(C,D)**. **(A,C)** depict individual samples while **(B)** and **(D)** show the taxonomic profiles according to site/habitat.

**FIGURE 4:**
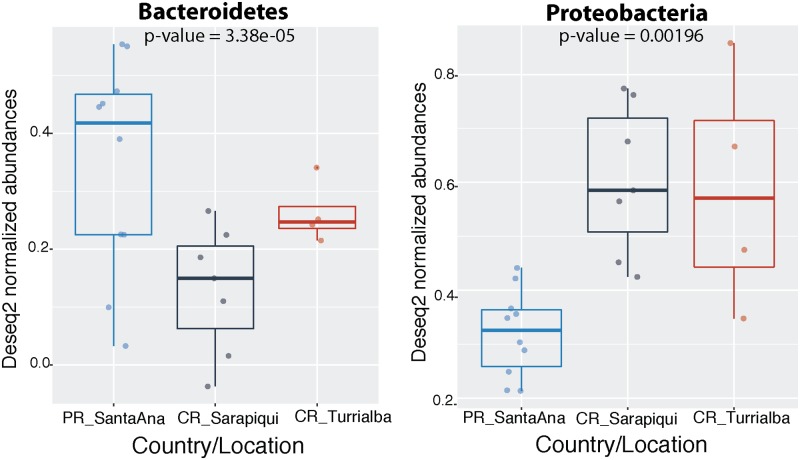
Significantly different phyla among the two countries. Abundances were normalized through DESeq2 negative binomial Wald normalization.

As discussed before, the dominating phyla were Proteobacteria, Bacteroidetes, Actinobacteria, and Firmicutes (**Figure 3**). Interestingly, we found that the Puerto Rican samples were significantly dominated by Bacteroidetes (ANOVA, *df* = 2, *F*-value = 19.25, *p*-value = 3.38*e*-05) while Costa Rican samples were dominated by Proteobacteria (ANOVA, *df* = 2, *F*-value = 8.99, *p*-value = 0.00196) (**Figure 4**). Regarding both Costa Rican sites, the most notorious difference at phylum level is that in Sarapiquí there is a higher abundance of Proteobacteria, Firmicutes, and Cyanobacteria compared to Turrialba (**Figure 3B** and **Supplementary Figure S1**).

At the genus-level, *Niabella* OTUs were highly dominant in Puerto Rico (∼25%) and the third most abundant in the two sites in Costa Rica. *Halomonas* OTUs had higher abundances in Sarapiquí (∼31%) compared to Santa Ana (<0.001%) and Turrialba (0.001%). *Bacteroides* OTUs were dominant in Turrialba samples (∼13%), as compared to Sarapiquí (0.006%) and Santa Ana (0.004%) (**Figures 3C,D**). Tables representing the relative abundance values for each sample at the phyla and genus levels can be found in the **Supplementary Tables S5**, **S6**.

**FIGURE 5:**
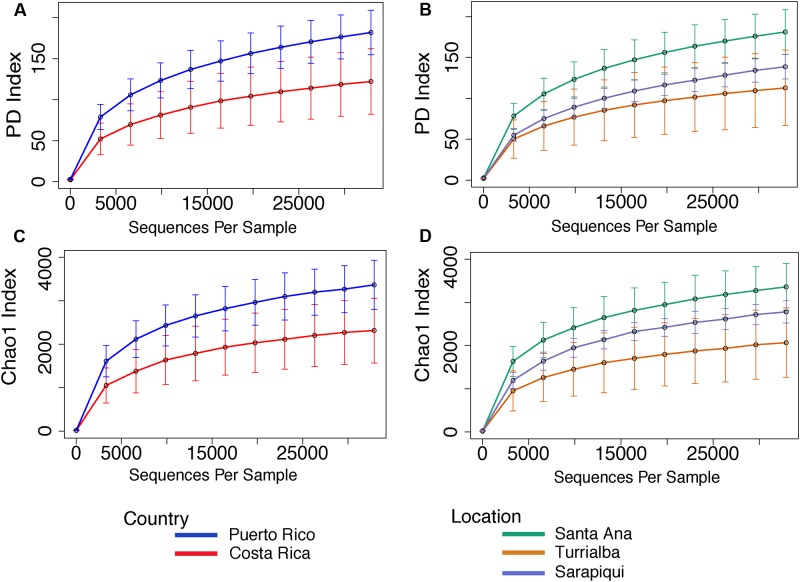
Rarefaction curves for alpha diversity measures of Faith’s PD index **(A,B)** and Chao 1 richness index **(C,D)** comparing country and location. Error bars in the figures correspond to one standard deviation out from the average (*n* = 10 biological replicates in Puerto Rico/Santa Ana; *n* = 7 biological replicates Sarapiquí, and *n* = 4 biological replicates Turrialba). PD measures per comparing countries indicate a significantly higher diversity in Puerto Rico (*t*-test, *t*-stat = 3.621, *p*-value = 0.004). Comparisons per location indicate that Santa Ana (PR) has significantly higher diversity compared to Sarapiquí (CR) (*t*-test, *t*-stat = –3.594, *p*-value = 0.021). Richness was significantly higher in Santa Ana compared to Sarapiquí (*t*-test, *t*-stat = –3.714, *p*-value = 0.009) but not compared to Turrialba (*t*-test, *t*-stat = –1.883, *p*-value = 0.291). Rarefaction analyses were based on 32,900 sequences per sample type.

The microbiota from Puerto Rican toads is significantly more diverse than the microbiota from Costa Rican toads (*t*-test, *t*-stat = 3.621, *p*-value = 0.004), as is its Chao 1 richness (*t*-test, *t*-stat = 3.723, *p*-value = 0.002) (**Figure 5** and **Supplementary Table S4**). As for the habitat/site, we found significant differences in diversity between the three locations (*p*-value = 0.01031). Nonetheless, pairwise comparisons showed that diversity was significantly different between Santa Ana and Sarapiquí (*t*-test, *t*-stat = -3.594, *p*-value = 0.021), as was richness (*t*-test, *t*-stat = -3.714, *p*-value = 0.009) (**Supplementary Table S4**).

**FIGURE 6:**
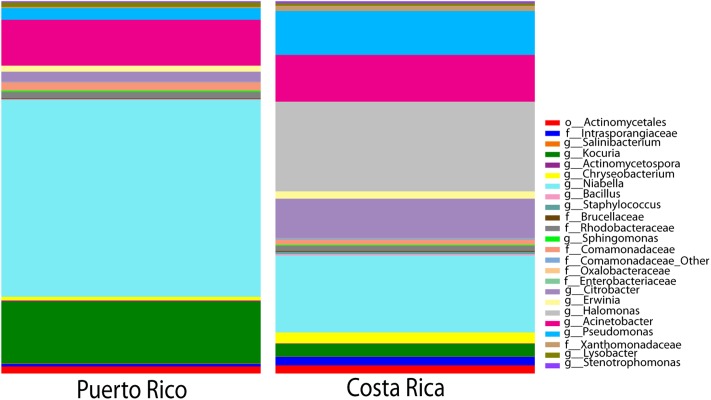
Taxonomic profile of core OTUs. Includes only OTUs present in 100% of samples both in Costa Rica as well as in Puerto Rico. The number of OTUs shared across 100% of the samples in both countries is 128 OTUs out of the original 5,152 (∼2.5% core species).

Core diversity analyses between toads in the two countries interestingly revealed that 128 OTUs were shared across all 21 toads (100% samples) (**Figure 6**). At the genus level these 128 OTUs represent 24 different genera, these include a dominance of *Halomonas*, *Pseudomonas*, and *Acinetobacter* in Costa Rica, and the expected *Niabella* in the Puerto Rican samples (**Figure 6** and **Supplementary Table S7**).

**FIGURE 7:**
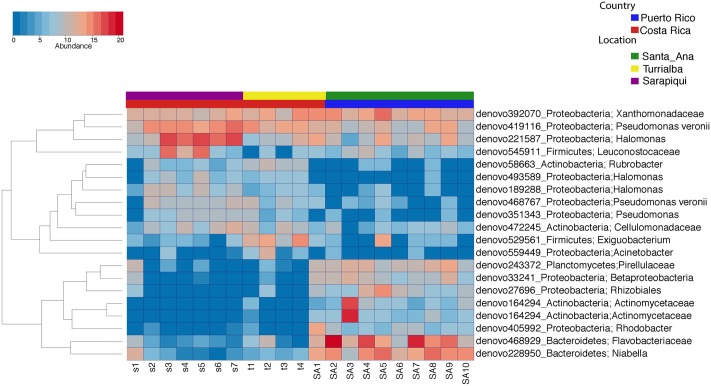
Heatmap showing the significantly different taxa among country and location/habitat according to a parametric log-likelihood ratio test (*p* < 0.05).

**FIGURE 8:**
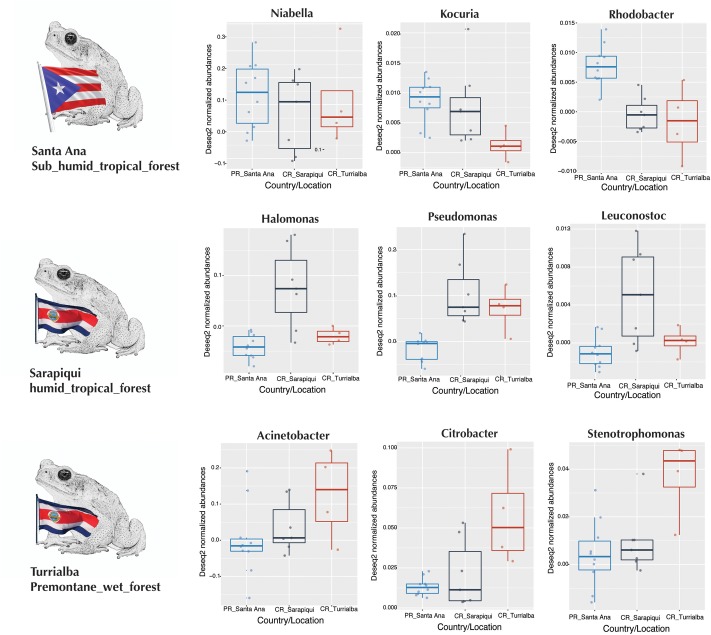
Boxplots of taxa differentially abundant at each country/location.

We then proceeded to determine which taxa changed significantly (selected OTUs with FDR *p ≤* 0.05) between the two countries and the three locations/habitats, by employing a log-likelihood ratio test. Significantly different taxa between countries resulted in 20 OTUs, most remarkably an abundance in *Niabella* and Flavobacteriaceae in Puerto Rico, and a dominance of *Halomonas* in Costa Rica (**Figures 7**, **8**). In fact, *Halomonas* was significantly abundant in Sarapiquí as was *Pseudomonas* and *Leuconostoc*, while *Acinetobacter* and *Citrobacter* were highly abundant in Turrialba (**Figures 7**, **8**).

## Discussion

Capitalizing on advances in next-generation sequencing, several recent studies on amphibian skin microbiota have revealed the importance of cutaneous microbes for host disease resistance (Kueneman et al., 2014; Walke et al., 2015; Rebollar et al., 2016b; Bletz et al., 2017b). This is the first report of the microbiota of the successful toad colonist *R. marina* highlighting differences between habitats where animals are indigenous (two locations in Costa Rica) and those where it is invasive (Puerto Rico). Given that we had a small sample number at each location and only two countries were compared, we will limit the discussion to geographical differences and the possible effects of habitat and environment. Overall, many genera found in this study correspond to previous reports in other bufonids. In fact, *Pseudomonas*, *Sphingobacterium*, and *Bacteroides* were the most common genera found in the western toad, *Anaxyrus boreas* (Kueneman et al., 2014), while *Pseudomonas*, *Acinetobacter*, *Pantoea*, and *Chryseobacterium* were the most important genera in *Bufo japonicus* (Sabino-Pinto et al., 2016, 2017). All these genera, except *Pantoea*, were represented in the *Rhinella* microbiota.

Microbial symbioses have been considered a foundational principle for the invasive success of several different species. Microbiomes enhance the capability of species to adapt to new niches as was first reported by a large mammalian study (Ley et al., 2008), as well as in other non-mammalian cases including insects (Engel and Moran, 2013), fish (Ye et al., 2014), amphibians (Kohl et al., 2013), and even plants (Bulgarelli et al., 2013; Coats and Rumpho, 2014). We found that alpha diversity measures were significantly higher in Puerto Rico where *R. marina* toads were introduced, compared to the two locations in Costa Rica (native range), but these differences may be driven by the environmental differences of the habitats. Interestingly, a similar pattern was found in plant bacterial communities, where native plants shown to have lower microbial species diversity and increased abundance of pathogens compared to their invasive counterparts (Coats and Rumpho, 2014). The high diversity in the Puerto Rican samples may be related to a number of factors including environmental or genetic factors associated with different populations as seen in other amphibians (Kueneman et al., 2014; Rebollar et al., 2016b). A higher diversity in the Puerto Rican frogs (those in the native range) may provide the host with a plethora of antimicrobial peptides, and the capacity to use resources more efficiently than communities with low species richness in the native range.

Like plant roots, the toad skin surface is in close contact with the environment, mainly with soil and water; therefore, it would not be surprising to find microbial communities in frog skin to have similar patterns as those of plants in introduced environments. Interestingly, statistical tests on beta diversity confirm significant differences between toad microbes in the two geographies, similar to the separation between microbiota of frogs from tropical and temperate zones (Belden et al., 2015).

We also found a greater dispersion pattern in the microbiota of toads from Sarapiquí, a humid tropical forest. The complex conditions of the amphibian skin (pH and epithelial solutes) in the different locations may influence the structure of the microbiota, as animals from Sarapiquí have higher pH and communities are distant. Although the impact of host factors on the skin microbiota is acceptable, it is still poorly understood how environmental factors influence the biogeographic patterns of microbial communities in amphibians, which may be due to precipitation or even nitrogen deposition in these tropical ecosystems (Hietz et al., 2011).

Cane toads are very effective invaders and very resistant to adverse conditions (Solís et al., 2009) and infections (Lips et al., 2006). Resistance can occur, among other reasons, by the presence of beneficial bacteria in the skin of amphibians (Madison et al., 2017). Interestingly, some of the bacteria we found in these toads including genera like *Acinetobacter* and *Pseudomonas* in Turrialba and *Kocuria* or *Chryseobacterium* in Puerto Rico were reported to inhibit the pathogen *B. dendrobatidis* (Holden et al., 2015). The diversity of the microbial communities could be indicative of invasive success, however, because only three populations and two countries were compared, we recognize that more extensive sampling of individuals in different locations within both countries is needed to corroborate this trend.

Previous studies on amphibian microbes have shown that host species is a greater predictor of bacterial communities than habitat (McKenzie et al., 2012), however, it has also been shown that similar composition occurs at high taxonomic levels such as Phyla with only differences at the genus and species levels (Belden et al., 2015; Rebollar et al., 2016b).

The Cane Toad *R. cf. marina* besides having marked differences in structure between the two countries it also exhibits a core microbiome composed by 128 OTUS. Genera shared among all samples in both countries included *Niabella*, *Kokuria*, *Pseudomonas*, *Acinetobacter*, and *Chryseobacterium*, and this may be an indicator of a strong symbiotic relationship with this amphibian species, although more in-depth studies may be needed across several geographic regions to confirm this hypothesis. In fact, like the NMDS patterns of the current study, microbial communities in Panamanian frogs revealed different clusters according to sampling site (Belden et al., 2015). The Panamanian frog model has also showed that besides transient bacteria, there is a species-specific microbiota and the more distant bacterial communities correspond to samples infected with *Bd* (Rebollar et al., 2016b). Likewise, and regardless of its core microbiome, cane toads exhibit abundance-specific OTUs at each location such as *Niabella* and *Kocuria* in Puerto Rico, *Halomonas* in Sarapiquí, and *Acinetobacter* in Turrialba. Bacterial genera that have been associated with improved host defense against pathogens in other amphibian studies include *Pseudomonas*, *Acinetobacter*, *Stenotrophomonas*, and *Chryseobacterium* (Flechas et al., 2012), all of them are present in the core microbiome of cane toads from both countries. Some genera such as *Acinetobacter* are present at a similar relative abundance in both countries, while others, such as *Pseudomonas*, are more dominant in Costa Rica.

*Niabella* is the most dominant genus in the *Rhinella* population of Puerto Rico being shared by all Puerto Rican samples and the second most dominant taxa in Costa Rica, to our knowledge this is the first report of this bacteria symbiotically associated at high dominance with an amphibian. These are Gram-negative bacteria, aerobic, non-flagellated, and rod-shaped and they produce flexirubin-type pigments (Dai et al., 2011). There are seven species described (Glaeser et al., 2013) isolated from soils (Dai et al., 2011; Ngo et al., 2017), water (Siddiqi and Im, 2016) medicinal leeches (Kikuchi et al., 2009), as well as epiphytic communities in the green macroalgae *Cladophoraglomerata* (Zulkifly et al., 2012). This bacterium was indeed found associated with leeches and macroalgae, both highly humid environments, just like the toad skin. In fact, leeches are common in pathogenic or phoretic associations with amphibians (Stead and Pope, 2010; Maia-Carneiro et al., 2012). This is the first report of *Niabella* in association with a new world amphibian and its high dominance warrants further studies.

*Halomonas* is another bacterial genus worth discussing due to its high abundance in Costa Rica (mainly in Sarapiquí). Sarapiquí samples corresponded to young adults, compared to all the rest of the sampled toads both in Costa Rica and Puerto Rico and an ontogenic relationship of the frog skin microbiota has already been reported (Kueneman et al., 2014; Longo et al., 2015; Sabino-Pinto et al., 2017). Additionally, a comparison between adult and juvenile *Eleutherodactylus coqui* in Puerto Rico found that juveniles had a more diverse microbiota than adults, and certain OTUs present in juveniles were not found in adults (Longo et al., 2015). It is also possible that the habitat where these juveniles were captured could have influenced the microbiota of these young adults, such as debris and cellars. Cane toads have been identified as being capable of tolerating highly saline environments in the wild (De León and Castillo, 2015). In fact, *Halomonas* have been isolated from saline environments (Sorokin and Tindall, 2006), rhizosphere (Borsodi et al., 2015), and have also been associated with rodents (Gavish et al., 2014). More studies comparing the skin microbiota of the cane toad at different stages of development should be done to further understand the type of association between *Halomonas* and this amphibian host.

The appearance of a new species in an ecosystem greatly impacts local diversity as already well described with the introduction of the pathogen *Bd* in frogs worldwide (Borzee et al., 2017) nonetheless, other animals such as geckos can bring different varieties of pathogenic bacteria (Gugnani et al., 1986) or parasites to the regions where they are introduced (Kelehear et al., 2015). Usually these risks are not well measured because the introductions are not controlled or monitored; therefore, next-generation sequencing tools take a special importance in the prevention of introduction of pathogens. In fact, amphibian microbiome studies have been increasing in recent years due to concerns about the disappearance of amphibians (Rebollar et al., 2016b; Jiménez and Sommer, 2017).

To our knowledge, this is the first study conducted to determine differences in skin microbiota between cane toads in two different geographical regions corresponding to exotic and native ranges. Our study confirms both the existence of bacterial OTUs composing a core microbiota in the *R. marina* sampled individuals, location-based patterns with significantly different taxa and reveals dominance of taxa such as *Niabella*, for the first time associated to the amphibian skin. We believe, therefore, that further sampling across global geographies in the native and exotic ranges are needed to further understand the microbial ecology of this species and to obtain a better understanding of the relationships between the microbiota in invasive species, likely leading to new insights into what microbes deem a successful invasion and allow the design of new microbiome-based control approaches.

## Author Contributions

JA conceived and designed the experiments, performed the experiments, analyzed the data, wrote the paper, prepared the figures and/or tables, and reviewed drafts of the paper. IZ and GO-M performed the experiments, analyzed the data, prepared the figures, and reviewed drafts of the paper. AL, MV-C, and NR-H performed the experiments, analyzed the data, and reviewed drafts of the paper. FV-S and CM-C performed the experiments and reviewed drafts of the paper. ET-R performed the experiments, contributed with materials, reviewed drafts of the paper, and funding. AP-T conceived and designed the experiments, performed the experiments, wrote the paper, reviewed drafts of the paper, and funding. FG-V conceived and designed the experiments, performed the experiments, analyzed the data, contributed reagents/materials/analysis tools, wrote the paper, prepared the figures and/or tables, reviewed drafts of the paper, and funding.

## Conflict of Interest Statement

The authors declare that the research was conducted in the absence of any commercial or financial relationships that could be construed as a potential conflict of interest.

## Footnotes

1 http://www.earthmicrobiome.org/emp-standard-protocols/16s/

## Supplementary Material

**FIGURE S1 |** Most abundant phyla across the three sampling locations. Abundances were normalized through DESeq2 negative binomial Wald normalization.

**TABLE S1 |** Total number of sequences and Operational Taxonomic Units (OTUs) for the 42 samples.

**TABLE S2 |** Total number of sequences and OTUs for the 21 collapsed samples.

**TABLE S3 |** Operational Taxonomic Unit table.

**TABLE S4 |** Statistical analyses comparing metadata, beta diversity and alpha rarefaction values for country, habitat and body site using Student’s *t-*test.

**TABLE S5 |** Relative abundance for each Phyla-level OTU for each of the three habitats.

**TABLE S6 |** Relative abundance for each genus-level OTU for each of the three habitats.

**TABLE S7 |** Core OTUs shared by the 21 toad samples.
