# Supplementary figures and images for: Characterization of the Skin Microbiota of the Cane Toad Rhinella cf. marina in Puerto Rico and Costa Rica
Source: Front Microbiol. 2018 Jan 5;8:2624. doi: 10.3389/fmicb.2017.02624 (PMC5760547; doi:10.3389/fmicb.2017.02624)

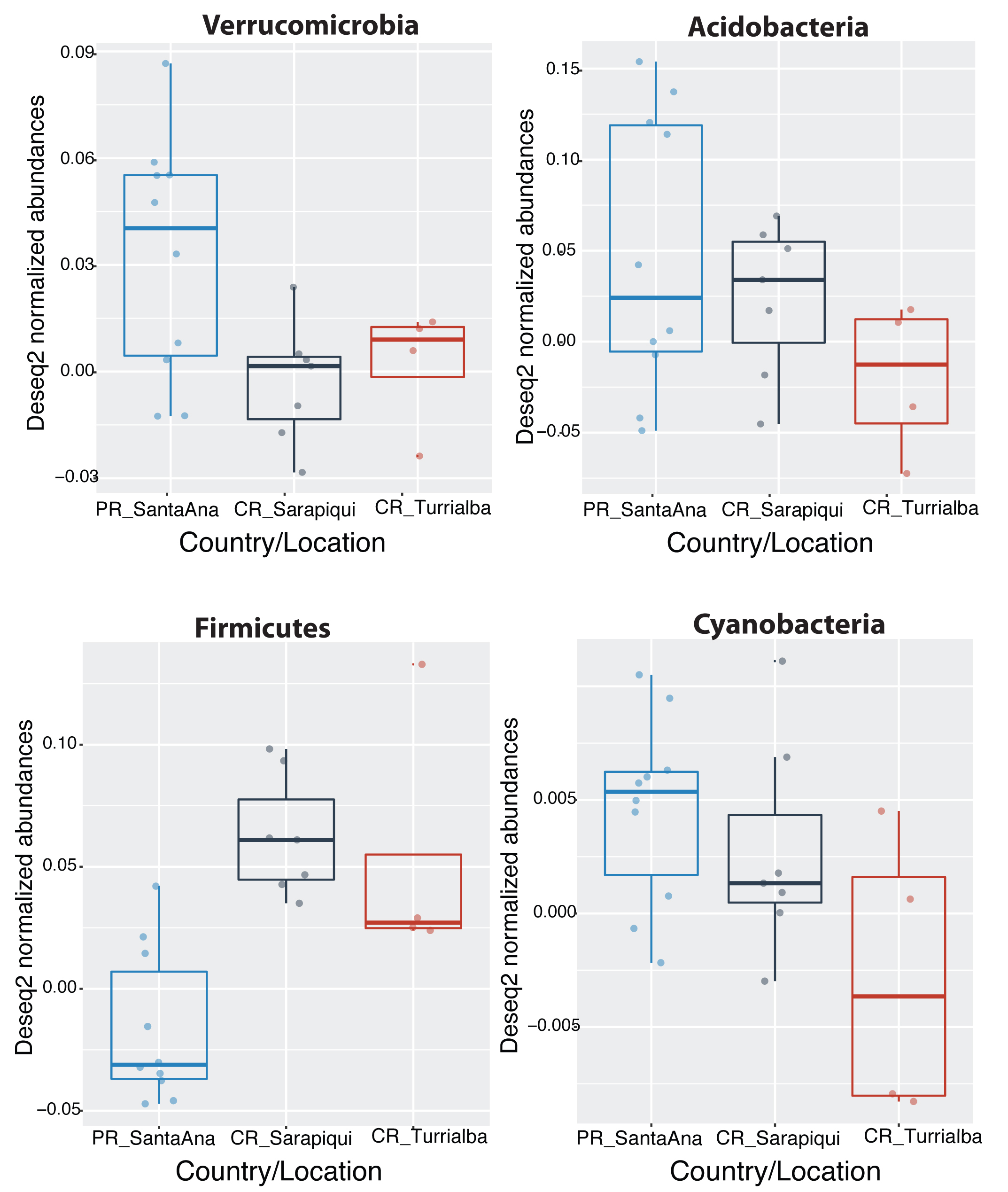

Supplement: Supplementary file 1 [file Image_1.JPEG]
